# Void Hole Avoidance for Reliable Data Delivery in IoT Enabled Underwater Wireless Sensor Networks

**DOI:** 10.3390/s18103271

**Published:** 2018-09-28

**Authors:** Arshad Sher, Aasma Khan, Nadeem Javaid, Syed Hassan Ahmed, Mohammed Y Aalsalem, Wazir Zada Khan

**Affiliations:** 1 Department of Computer Science, COMSATS University Islamabad, Islamabad 44000, Pakistan; arshadsher92@gmail.com (A.S.); aasmakhan749@gmail.com (A.K.); 2 Department of Computer Science, Georgia Southern University, Statesboro, GA 30460, USA; s.h.ahmed@ieee.org; 3 Farasan Networking Research Laboratory, Department of Computer Science & Information System, Jazan University, Jazan 82822-6694, Saudi Arabia; aalsalem.m@jazanu.edu.sa (M.Y.A.); wazirzadakhan@jazanu.edu.sa (W.Z.K.)

**Keywords:** underwater wireless sensor networks, adaptive transmission range, residual energy, clustering, void hole, collision

## Abstract

Due to the limited availability of battery power of the acoustic node, an efficient utilization is desired. Additionally, the aquatic environment is harsh; therefore, the battery cannot be replaced, which leaves the network prone to sudden failures. Thus, an efficient node battery dissipation is required to prolong the network lifespan and optimize the available resources. In this paper, we propose four schemes: Adaptive transmission range in WDFAD-Depth-Based Routing (DBR) (A-DBR), Cluster-based WDFAD-DBR (C-DBR), Backward transmission-based WDFAD-DBR (B-DBR) and Collision Avoidance-based WDFAD-DBR (CA-DBR) for Internet of Things-enabled Underwater Wireless Sensor Networks (IoT, UWSNs). A-DBR adaptively adjusts its transmission range to avoid the void node for forwarding data packets at the sink, while C-DBR minimizes end-to-end delay along with energy consumption by making small clusters of nodes gather data. In continuous transmission range adjustment, energy consumption increases exponentially; thus, in B-DBR, a fall back recovery mechanism is used to find an alternative route to deliver the data packet at the destination node with minimal energy dissipation; whereas, CA-DBR uses a fall back mechanism along with the selection of the potential node that has the minimum number of neighbors to minimize collision on the acoustic channel. Simulation results show that our schemes outperform the baseline solution in terms of average packet delivery ratio, energy tax, end-to-end delay and accumulated propagation distance.

## 1. Introduction

Underwater Wireless Sensor Networks (UWSNs) have attracted both academia and industry to explore the underwater resources by enabling a variety of aquatic applications. For instance, military defense, monitoring the aquatic environment, disaster prevention, pollution monitoring, underwater mineral extraction, etc. [1]. The sensor nodes are randomly deployed over a specified geographic volume with the ability to sense, gather and transmit data towards the destined location (that may be a single sink or multiple sinks) [2,3].

Data communication in the acoustic medium faces several challenges due to the peculiar features of the aquatic environment like high propagation delays, high deployment cost, node movement due to water currents, energy constraints, limited bandwidth, etc. [4,5]. Various routing protocols are proposed to enhance the network lifetime with optimal energy consumption and minimize delay from the source to the destination using direct or multihop data transmission mechanisms [6,7,8]. To deliver the data to the destination, geographic routing is widely used for both aforesaid data communication methods depending on the nature of the environment. Geographic routing uses the greedy forwarding strategy where each node finds the shortest path towards the destination to save its energy. However, in the greedy forwarding strategy, immutable forwarder node selection is inevitable, which leads to immature depletion of the node’s battery and creates a void hole [9]. The void hole is avoided using the Adaptive Hop-by-Hop Vector-Based Forwarding (AHH-VBF) routing protocol [1]. It uses a pipeline to restrict the transmission range, and it is adaptively adjusted to amend the forwarding area to reduce duplicate packets’ transmission.

However, the void occurrence is not avoided in sparse deployment using this algorithm [9], because it reacts once the data packet is trapped and the data communication process is paused. This may occur because of the variation in the path quality, which is important for energy efficiency in IoT-enabled WSN. The major factors to be considered in the path to avoid void occurrence are: shortest distance and lesser number of links to enhance the network lifetime [10,11]. The IoT-enabled WSN has the ability to sense, gather and transmit a huge amount of data over a long distance. However, the limited batteries are the major hurdle in successful network operations. Thus, we need to schedule the data transmissions in energy constraint networks. Similarly, the network virtualization is another important aspect to find the path fault successfully in IoT-enabled WSN. The virtualization focuses on the optimal utilization of sensing resources. Moreover, it also supports diversity in the network and enables an efficient management of power resources. However, it has a reactive approach in handling the link failure [12]. The IoT-enabled WSN has been helpful in connecting anything, anywhere. Anywhere means sensors, vehicles, cameras, watches, phones, etc. [13]. The vehicles with customized sensors can allow communication with nearby IoT-enabled WSN. In fact, vehicles are feasible for communication because they do not have the energy limitation problem. However, our focus is on the energy constraint in IoT networks in which the device battery needs to be efficiently utilized. Thus, we need to have a routing algorithm that takes precautionary measures in advance to save data packet loss and handle the occurrence of a void node efficiently while saving the node’s energy [14]. Therefore, to achieve energy efficiency along with minimum delay in IoT-enabled UWSNs, the need for robust routing algorithm emerges, which can be adapted according to the available resources.

In this paper, we propose four schemes: Adaptive transmission range-based WDFAD-Depth-Based Routing (DBR) (A-DBR) and Backward transmission-based WDFAD-DBR (B-DBR) are proposed to reduce the probability of void hole occurrence. While the A-DBR scheme adjusts its transmission range to overcome the void hole problem to continue the greedy forwarding of the data towards the sink, B-DBR exploits a fall back recovery mechanism to find out an alternative route for delivering data at the destination. Additionally, we propose Cluster-based WDFAD-DBR (C-DBR) to minimize end-to-end delay and reduce energy consumption. The Collision Avoidance-based WDFAD-DBR (CA-DBR) handles the collision problem by selecting a potential forwarder node with the minimum number of neighbors. The main scientific contributions of this paper are:

- Two techniques, A-DBR and B-DBR, are proposed to avoid void holes.
- Two techniques, CA-DBR and C-DBR, are proposed to avoid collision and minimize the packet drop ratio.
- C-DBR selects the forwarder node with the maximum residual energy in order to enhance the lifetime of the network.
- The proposed schemes are compared with WDFAD-DBR in terms of average packet delivery ratio, energy tax, end-to-end delay and accumulative propagation distance.

The rest of this paper is organized as follows: In Section 2, related work on existing schemes in UWSNs and the problem statement are presented. In Section 3, the background is discussed including the system model, energy consumption and propagation models. Section 4 describes the proposed schemes in detail. Simulation results are presented in Section 5. Performance trade-offs are given in Section 6, followed by the conclusion in Section 7.

## 2. Related Work and Problem Statement

The proliferation of sensing devices has enabled real-time monitoring through WSNs. These devices are low cost and can easily be deployed to gather data from the region of interest. In this perspective, UWSN has emerged to provide a feasible surveillance system for the rich resource of the acoustic environment. Therefore, the research community wants to explore underwater resources; however, an efficient routing algorithm that can provide reliable communication is desired, such as AHH-VBF, which is used to reduce energy consumption by adjusting the range of the forwarding vector [1]. To reduce energy consumption and avoid void hole occurrence, it dynamically adjusts the transmission power at each hop along with the vector. However, with the adjustment in transmission power, the energy dissipation increases.

A GEographic and opportunistic routing with Depth Adjustment-based topology control for communication Recovery over void regions (GEDAR) is proposed in [15]. It uses the greedy routing strategy to forward packets towards the sink node. Moreover, a priority is assigned to each neighbor node to avoid redundant transmissions by only allowing the highest priority node to transmit the data. In case the transmission fails, the node with low priority in the table resumes transmission from an alternate route. Additionally, this protocol uses a depth adjustment mechanism to provide continuous communication among the network nodes. However, moving nodes to a new depth causes excessive energy consumption and high end-to-end delay.

The Hydraulic-pressure-based anyCast (HydroCast) [16] algorithm was designed to deliver data reliably to any sink positioned at the surface of the water. The forwarder node is chosen on the basis of the packet status and the cost of the link. Through a gauge, the depth information is obtained for successful data transmissions. This scheme has improved Packet Delivery Ratio (PDR) due to the low ratio of void node occurrence at the cost of high communication overhead, which causes more energy depletion of the network nodes.

In [17], the authors proposed the Hop-by-Hop Dynamic Addressing-based Routing Protocol for Pipeline Monitoring (H2-DARP-PM). Dynamic hop addresses are assigned to each hop to enable efficient forwarder node selection. This scheme assigns dynamic a hop address to every node that contributes to data forwarding. This scheme improves the PDR; however, the energy consumption is considerably high.

Delay-sensitive schemes: Advancement of localization-free routing protocols of DBR, EEDBRand AMCTD [18] are presented for time-critical applications. The authors have made these routing protocols scalable according to the application requirements to achieve minimum end-to-end delay along with the minimal energy dissipation. However, duplicate packets are forwarded very often because of the hidden terminal problem. In delay-sensitive EEDBR, the energy consumption is high, whereas the packet drop ratio is considerably improved in AMCTD.

Free Space Optical (FSO) and Electro Magnetic (EM) wave-based communication schemes [19] have been used to examine an analytical framework to find an optimum range of clusters. Moreover, the logical results are computed to change the location of the sink to three different points: the center, corners and midpoint of the network field. This scheme results in less energy consumption at the cost of high end-to-end delay.

To save energy, sleep-awake scheduling is a widely-accepted mechanism. For instance, in [20], the authors nominated an initiator node after the configuration of network nodes to gather data from the desired nodes. The communication phase begins with the initialization of the transmission phase. First of all, a head node is selected at each hop to lead the data packet towards the destination. Only the head node transmits the data, and nodes in the neighborhood are switched to sleep mode to avoid the unnecessary dissipation of the node’s battery. This scheme reduced the energy consumption significantly with enhanced lifetime and increased PDR. However, the immutable selection of the head node results in the sudden death of the node and degrades the network performance.

To collect data at distributed points, clustering is performed because it is scalable and flexible in nature. The same features motivate the research community to explore this area in more detail. In [21], the network was divided into irregular clusters for making local routing decisions to avoid high data traffic at the sink node. Moreover, the algorithm forms irregular clusters based on a layered architecture for event coverage and obtains the expected value of the clusters through theoretical analysis. Additionally, this scheme uses a recovery strategy to balance the energy consumption among the clusters to enhance the performance of the network.

A Particle Swarm Optimization-based Energy-efficient Cluster Head Selection (PSO-ECHS) was proposed in [22]. To balance the energy consumption, various control parameters were taken into the consideration like distance within the clusters and from the sink along with the residual energy of each node in the cluster. With the help of the aforesaid metrics, a weighting function was formulated, and probabilistic value was computed to nominate and rotate the head node for efficient energy consumption. This scheme achieves high PDR at the cost of delay.

In [23], three schemes were proposed: Sparsity-Aware Energy-Efficient Clustering (SEEC), Circular SEEC (CSEEC) and Circular Depth-based SEEC (CDSEEC). In SEEC, two mobile sinks are deployed in sparse and dense regions to collect information and to reduce the probability of energy hole occurrence; while a different geometry is considered for CSEEC to analyze the mobility of sinks, which improves the PDR and maximizes the network lifetime. The same topology is considered for CDSEEC with a different mobility pattern. The trade-off occurring against energy efficiency and PDR is the highest end-to-end delay.

Depth-Based Routing (DBR) [24] uses a greedy approach to deliver packets towards the sink based on the depth of a forwarder node. Each eligible source node transmits a packet based on depth and also calculates the holding time to avoid duplicate packets’ transmission among the network nodes. However, the consideration of only distance in the selection of the next hop node forces the immutable nomination of the forwarder node. This leads to sudden death of the intermediate nodes. Moreover, the holding time is not synchronized, resulting in transmission from the neighbor nodes before even the acknowledgment arrives. However, DBR benefits from high network lifetime and PDR at the cost of only end-to-end delay.

An improved Adaptive Mobility of Courier nodes in Threshold-optimized DBR (iAMCTD) [25] is presented to handle flooding, latency and path loss. The routing is performed on demand to maximize the network lifetime through an optimized mobility pattern of courier nodes, whereas, the Energy-efficient Channel-Aware Routing Protocol (E-CARP) [26] provides improved network lifetime and reduced energy consumption by the reactive routing approach.

In Adaptive Relay Chain Routing (ARCR) [27], the authors introduced mobile sensor nodes to overcome the energy hole problem. Additionally, clusters were formed for collecting data via mobile nodes to improve the network performance. This routing mechanism achieved energy efficiency and maximum lifetime at the cost of low PDR.

The proposed work is different from the discussed related work based on the following distinguishing features. In order to reduce the probability of void hole occurrence, A-DBR adjusts its transmission range adaptively. However, the adjustment of transmission power causes extra energy consumption when the distance increases between the source and destination. Thus, B-DBR looks for a forwarder in all possible directions within its transmission range to find an alternate path to deliver data at the destination; while C-DBR and CA-DBR minimize end-to-end delay along with collision by making clusters in the network. The related work is summarized in Table 1.

### 2.1. Problem Statement

**Figure 1:**
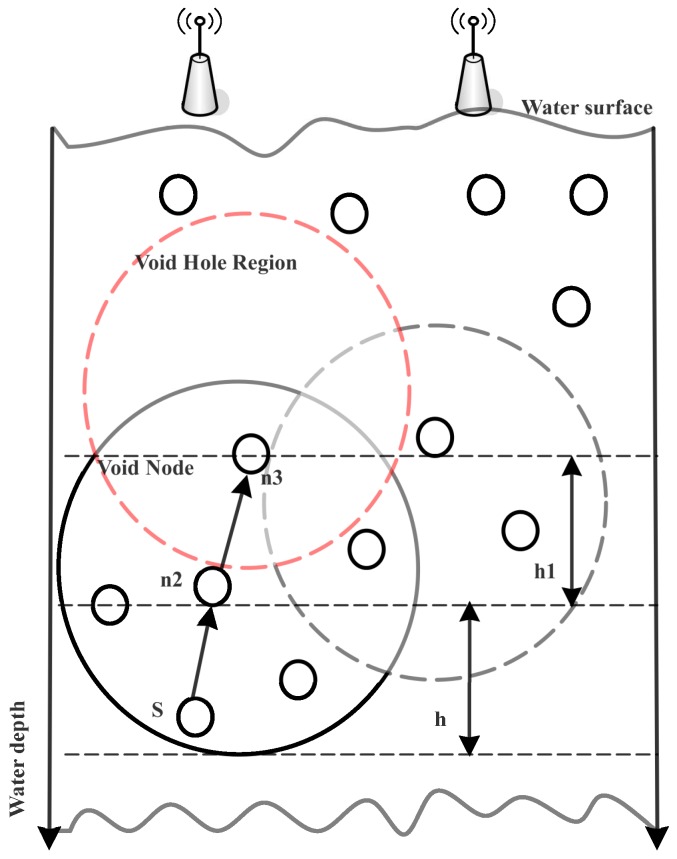
Illustration of the void hole problem in WDFAD-DBR.

To efficiently utilize the node battery and to reduce the end-to-end delay, the research community has been devoted to bringing improvement in routing algorithms designed for UWSNs. For instance, WDFAD-DBR [4] considers only two metrics: the depth of current and next expected forwarder node. Although, the probability of void hole occurrence is reduced and inefficient energy consumption during nodes communication is minimized, the probability of void hole occurrence still exists, as illustrated in Figure 1.

When the source node *S* initiates communication and finds S2 in its communication range, before transmitting the data packet to S2, it acquires information about its neighbor node. It locates S3 in its transmission range and delivers the data to S2. Thus, it acknowledges the *S* with non-void node status and receives the data packet. However, when S2 looks for its neighbors, it finds S3, which has no further nodes in its transmission range, resulting in loss of the data packet. Thus, this process will continue until the death of S3. Additionally, this scheme is receiver based, where avoidance of duplicate packets is very difficult. The reason is that neighbors in the hidden terminal region are unable to receive the acknowledgment, leading to redundant transmissions at the destination. Moreover, it also leads to channel interference in the case of simultaneous transmissions over the acoustic wireless channel. Furthermore, it causes collision, leading to a high packet drop ratio and more end-to-end delay. To overcome the aforementioned problems, we propose four schemes: A-DBR, C-DBR, B-DBR and CA-DBR, to improve the network performance. The details are given in the following sections.

## 3. Background

In this section, we discuss the system, energy and propagation models along with the type of packets used to configure the network. First of all, the system model is presented along with the assumptions made in the proposed work. Then, the energy and propagation models are briefly discussed followed by packet types.

### 3.1. System Model

**Figure 2:**
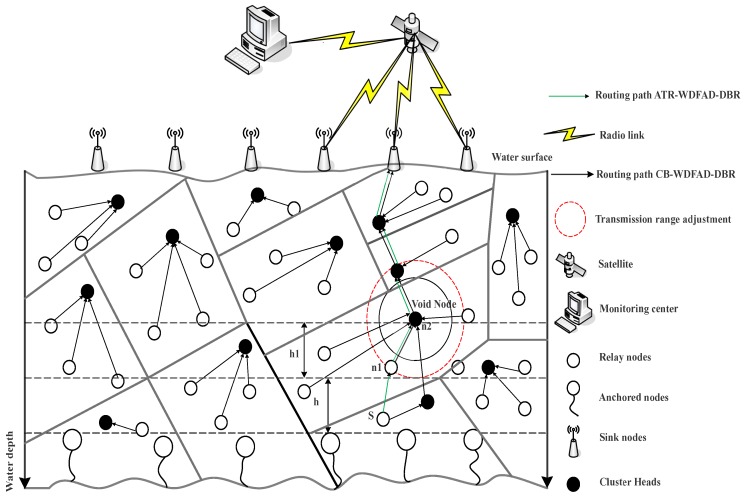
Proposed system model illustrating Adaptive transmission range in WDFAD-Depth-Based Routing (DBR) and Cluster-based WDFAD-DBR (C-DBR).

In proposed schemes, the 3D multi-sink network architecture is assumed [4], which is composed of the anchor, relay and sink nodes, as shown in Figure 2. The anchor nodes are fixed at the bottom of the water. These nodes are used to sense and to gather data, while the relay nodes are placed at different depths, which receive and forward the data towards the sink by collecting data from the anchor nodes. The sink node is housed with acoustic and radio modems to communicate with nodes deployed inside the water and in the terrestrial environment, respectively. Additionally, the burden of overhead is reduced by forming a cluster. It helps in local data gathering where only the head node of the cluster transmits data to the sink node. Additionally, it enables the network nodes to reduce the probability of interference via restricting the number of nodes involved in data transmission over the wireless acoustic channel. Furthermore, the selection of the Cluster Head (CH) is based on the residual energy, which allows continuous rotation of the head node. The sink nodes are placed at the surface of the water to direct data packets to the control center. An assumption is made that each sink is connected with the others through radio links. Furthermore, if a packet is received at one sink, it is assumed that data are successfully transmitted to the base station. The underwater settings affect the consumption of energy and the delay propagation of sound waves. To explain the underwater communication, Thorp’s propagation model is used [29].

#### 3.1.1. Energy Consumption Model

Underwater channel attenuation over a distance *l* can be demonstrated as [29]:

(1) 10logA(l,f)=c.10logl+l.10logα(f).

The first term of this equation represents the spreading loss, and the second term shows the absorption loss, where *c* is the spreading coefficient, which states the geometry of propagation, i.e., *c* = 1 is cylindrical spreading in shallow water, two is for spherical spreading in deep water and 1.5 depicts the practical spreading, where, α(f) is the absorption coefficient.

In UWSNs, the acoustic signal is affected by different noises, such as turbulence $N_{t}\left(f\right)$, shipping $N_{s}\left(f\right)$, waves $N_{w}\left(f\right)$ and thermal noise $N_{th}\left(f\right)$ [17,29,30,31]. These noises can be expressed as,

(2) $$N\left(f\right)=N_{t}\left(f\right)+N_{s}\left(f\right)+N_{w}\left(f\right)+N_{th}\left(f\right).$$

For the acoustic signal, the Signal to Noise Ratio (SNR) with frequency *f* and distance *l* can be expressed as:

(3) $$SNR\left(f,l\right)=T_{p}\left(f\right)-A\left(l,f\right)-N\left(f\right)+D_{i},$$

where $T_{p}\left(f\right)$ represents the transmission power with frequency *f*. $D_{i}$ denotes the directivity index to evade unnecessary noise. During the reception of an acoustic signal, if SNR(f,l) becomes greater or equal to detection threshold $D_{t}$, then the received signal is decoded correctly.

#### 3.1.2. Delay Propagation Model

Underwater delay propagation considers temperature, the depth of water and salinity of water, and it is given as follows [1]:

(4) $$\begin{array}{cc} \nu & =1448.96+4.591\tau -5.304\times 10^{-2}\tau^{2}+2.374\times 10^{-2}\tau^{3}\\ & +1.340\left(\delta -35\right)+1.63\times 10^{-1}d+1.675\times 10^{-7}d^{2}\\ & -1.025\times 10^{-2}\tau \left(\delta -35\right)-7.139\times 10^{-13}\tau d^{3}. \end{array}$$

Here, ν represents the propagation speed of the acoustic signal, which is measured in ms${}^{-1}$, τ represents the temperature, δ shows the salinity and *d* denotes the depth of water. The acoustic propagation speed is directly proportional to the temperature, salinity and depth of the water. Equation (4) is effective when it fulfills the conditions as: 0≤τ≤30, 30≤δ≤40 and 0≤d≤8000.

#### 3.1.3. Packet Types

In these schemes, there are three types of packets: namely $neighbor_{request}$, ack and $data_{packet}$. $neighbor_{request}$ consists of three fields: type ID, source node ID and depth. The type ID denotes the packet type; source ID represents the address of the source node; and depth illustrates the depth of the source node.

The ack packet has three data fields: type ID, source node ID and depth. The type ID represents the ack packet ID; source ID represents the source node ID; and depth is the depth of the source node.

On the other hand, $data_{packet}$ is composed of: type ID, source node ID, destination ID, depth and PID. The type ID, source node ID and depth represent the same as for the packet types $neighbor_{request}$ and ack. The destination ID represents the ID of the destination node, and PID (Packet ID) represents the order of packets.

$neighbor_{table}$ consists of the neighbor ID, depth, distance and time stamp. The neighbor ID indicates the location of the neighboring node. The distance shows the distance from the neighboring nodes. The time stamp represents the time for updating neighbors entry, and depth is the depth of the source node.

$packet_{queue}$ is generated to keep the record of all the sent and received data packets in order to restrain the replication of data packet transmission and for saving energy. The $Packet_{queue}$ consists of the fields of source ID, PID and flag. The source ID is the ID of the source node; PID is the Packet ID; and flag indicates whether the data packet has been sent or not.

#### 3.1.4. Explanation of Algorithm 1

Algorithm 1 describes the packet forwarding mechanism in general. First, node *i* receives a packet from node *j*. Then, it calculates the previous and current depths of the node. After calculating the depths of both nodes, node *i* calculates the distance difference of node *i* and the previous node corresponding to the Received Signal Strength Indicator (RSSI) of the Received Packet RSSI (RP-RSSI) and the RSSI of Signals at Senders (SS-RSSI). In these schemes, SS-RSSI is known to all nodes in the network. Therefore, every node in the network can calculate the distance between two nodes corresponding to Thorp’s propagation model. Next, the Relative Coordinate (RC) is calculated, corresponding to the depth difference between them.

**Algorithm 1** Algorithm for forwarding data packets Node *i* receives data_packet from node *j* Obtain $prev_{node}\_depth$ and $curr_{node}\_depth$ Calculate distance relative to diff(SS_RSSI,RP_RSSI) Calculate RC(sender,receiver) corresponding to distance and $diff(prev_{node}\_depth,curr_{node}\_depth)$ SWITCH (packettype) CASE 1: NeighborRequest **if** node *i* is the preferable forwarder node of node *j*
**then**  send ack **end if** Hold for next data packet END CASE CASE 2: Ack **if** node *j* is the preferable forwarder node of node *i*
**then**  up-to-date entry neighbor_tablemaking use of item ($prev_{node}\_depth,distance,t_{current}$) **end if** END CASE CASE 3: DataPacket Move to the next step END CASE END SWITCH **if** selected node *i* is not the preferable forwarder node of data_packet
**then**  Upgrade neighbor_table using item ($prev_{node}\_depth,t_{current},distance$)  Drop data_packet **end if** **if** node *i* is the preferable forwarder node of data_packet
**then**  Obtain source ID, packet ID from data_packet  **if** (source ID, packet ID) within the queue **then**   Drop data_packet  **end if**  **if** the node is within the forwarding area **then**   Move to the next step  **else**   Hold for the next data_packet  **end if** **end if** Find the next $depth_{min}$ in neighbor_table **if**
neighbor_table is empty **then**  Drop data_packet **end if** Upgrade the depth in data_packet with $current_{node}\_depth$ Add (source ID, packet ID) into the queue

The type of forwarded or received packets is checked accordingly. When a node receives the data packet, it manages the data packet as follows:

- If the node is not within the effective forwarding range of the preceding hop, it solely updates its neighbor table. In the other case, it enqueues information if it finds no report regarding the packet in the packet queue.
- If the node is not within the forwarding area, it will wait for the subsequent packet; this means that the packet is inside the suppression area. In any other case, the node searches for neighboring nodes in the neighbor table.
- If the neighbor table is empty, it will directly drop the data packet, as there are no nodes in the forwarding area. Thus, void holes can be prevented earlier.

## 4. Proposed Schemes

In this section, we describe the proposed schemes in detail. Every scheme has the same perquisites for configuring the network nodes. The forwarding mechanism of each scheme has been discussed as follows:

### 4.1. A-DBR

In this section, the proposed scheme is discussed in detail. We have proposed the A-DBR scheme to cater to the problems of the void hole discussed in Section 2.1. In this scheme, when a void hole occurs, it adaptively adjusts its transmission range and forwards the data packet towards the sink node, as shown in Figure 2. With a view to lessen neighborhood of requests, every node collects the records on neighbor nodes upon receiving packets: $neighbor_{requests}$ or ack. In this way, every node can reap newer statistics about neighbor nodes dynamically. As shown in Figure 2, in the A-DBR scheme, when node *S* senses data within its vicinity, it gathers the data packet and forwards it to nodes n1 and n2. Moreover, when a void node occurs, this scheme adjusts its transmission range as illustrated in Figure 2 to find a forwarder node and continues forwarding of information without dropping the data packet. In this scheme, when a node receives a packet, it handles the data packet as follows:

- If the node is not within the effective forwarding range of the preceding hop, it solely updates its neighbor table. In any other case, it enqueues information if it finds no report about the packets in queue.
- If the node is not within the forwarding area, it will wait for the subsequent packet, this means that the packet is inside the suppression area. In any other case, the node searches for nodes in the neighbor table.
- If the table is empty, instead of dropping a packet, the source node adjusts its transmission range and updates the neighbor table to avoid the void hole.
- After updating the neighbor table, it is going to send the packet if no different transmission of the data packet is heard towards the destination. It then updates the packet queue.

### 4.2. C-DBR

**Figure 3:**
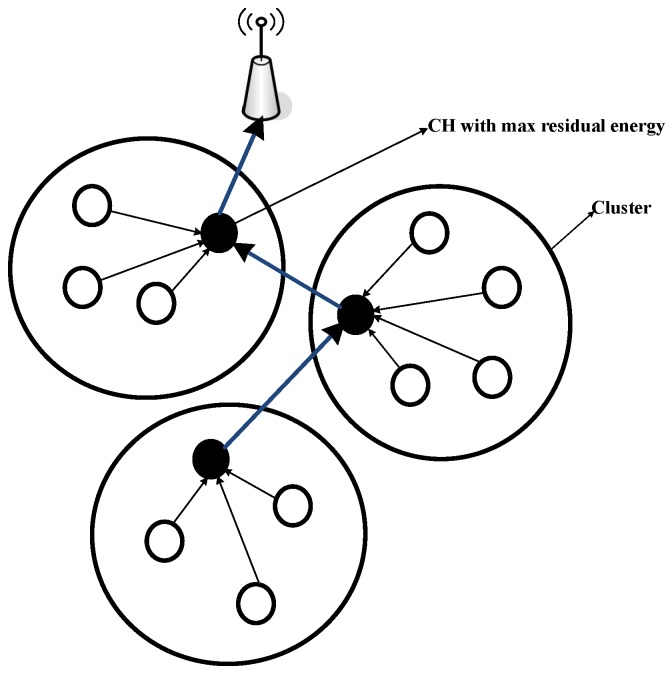
Illustration of cluster formation in C-DBR.

In C-DBR, we have formed clusters to restrict the access of the wireless channel by network nodes to avoid collisions. To select the CH, a node with maximum residual energy is nominated to avoid the immutable CH selection, which leads to high network lifetime. Additionally, it is assumed that the sink node has the knowledge of all the sensor locations. Further, the CHs are found by the source node based on the maximum residual energy. In C-DBR, node *S* forwards the sensed information towards the CH, which aggregates the neighbor packets to transmit the packet towards the immediate cluster’s CH near the destination. This process continues until it reaches the sink node, as shown in Figure 3. Below are the steps for selecting the CHs of C-DBR:

In this scheme, when a node receives a packet, it manages the data packet as follows:

- If the node is not within the effective forwarding range of a preceding hop, it solely updates its neighbor table without forwarding the packet. In any other case, it enqueues information if there is no report about the packet in the packet queue.
- If the node is not within the forwarding area, it will wait for the subsequent data packet; this means that the packet is inside the suppression area. Otherwise, the node searches for neighboring nodes in the neighbor table.
- Initially, there are no clusters in the network.
- The network is then divided into clusters using the k-means clustering approach.
- The source node broadcasts the message in the cluster.
- The sensor node then compares its own energy with the source node energy.
- If the sensor node energy is greater than the source node energy, then the sensor node sends a reply message; else, the source node waits for another reply from the neighbor node that has the maximum residual energy.
- Once the CHs are selected, clusters are formed using the neighbor nodes within the communication range.
- CH then broadcasts the message to the member nodes along with its ID to receive the data packets.
- CHs aggregates data to transmit a single data packet towards the sink directly or using the multi-hop forwarding approach.
- The neighbor table and packet queue are updated repeatedly till the death of all the network nodes.

### 4.3. B-DBR

**Figure 4:**
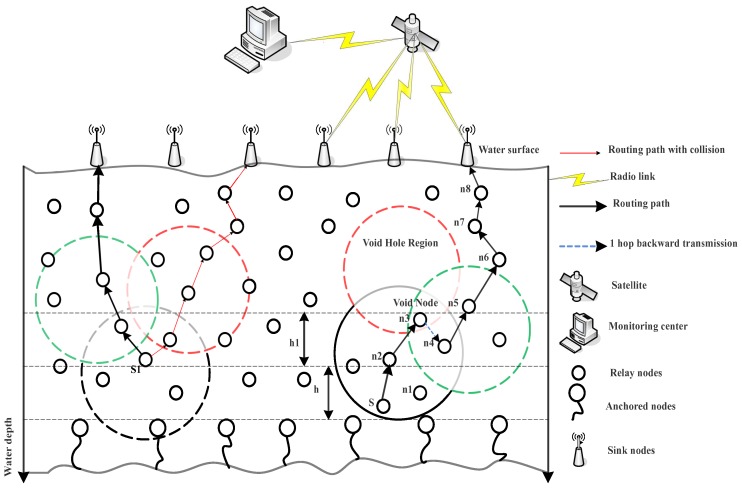
Proposed system model illustrating B-DBR and Collision Avoidance-based (CA)-DBR.

In this section, we describe the B-DBR routing protocol, which finds the set of forwarders at each hop using the greedy opportunistic forwarding mechanism. Additionally, it uses a fall back mechanism to find an alternative route to deliver the data in the case of the void hole region. On the right side of Figure 4 of B-DBR, the fall back approach is illustrated. When node *S* looks up two-hop neighbor information, there is the possibility of encountering a void node. In this case, B-DBR uses backward transmission from node n3 instead of depth adjustment, which consumes high energy. The node n3 forwards the data packet instead of dropping to node n4, which looks for its neighbors in the direction of the destination and finds nodes n5 and n6. Thus, greedy forwarding again is resumed till the time the packet reaches its destination.

#### Explanation of Algorithm 2

The main steps of the B-DBR protocol are represented in Algorithm 2. If a node is in the fall back recovery mechanism, a new data packet will be queued until the selected node in the backward direction has neighbors in the direction of the destination to resume the greedy forwarding mechanism. If it finds the forwarder nodes greater than zero, the data packet is forwarded. However, when the forwarder is not available, instead of dropping the data packet, it finds an alternate route to reschedule the data transmission. This process repeats itself until the death of all the nodes in the network.

**Algorithm 2** Main steps of the B-DBR scheme **if** void node or known sinks = 0 **then**  Queue the data packets  Re-schedule forward data packet() **else**  $f_{i}$ ⟵ get_next_hop_forwarder(n)  **if**
$|f_{i}|>0$
**then**   Forward the data packet  **else**   Queue the data packet   Re-schedule forward_data_packet()   Proposed_mechanism()  **end if** **end if**

### 4.4. CA-DBR

In this section, we describe the CA-DBR routing protocol, which also finds the set of next hop forwarders using the greedy opportunistic forwarding mechanism. CA-DBR selects those nodes that have the minimum number of neighbor nodes to avoid the collision, as shown on the left side of Figure 4. In this scheme, when a node receives a packet, it manages the data packet as follows:

- If the node is not within the effective forwarding range of the preceding hop, it does not forward the data packet, and it solely updates its neighbor table. Moreover, it enqueues information if the packet has not been transmitted already.
- Whereas, if the node is not within the transmission range of the forwarding node, then it will wait for the subsequent data packet. This means that the packet is inside the suppression area. This will reduce the collision and interference on the acoustic channel.
- The fall back and nomination of forwarder node mechanisms are used together for minimal energy consumption and a high packet delivery ratio.
- After updating the neighbor table, if no different transmission of the packet is heard towards the destination, it forwards the packet towards the sink node.

## 5. Linear Programming-Based Mathematical Formulation

Linear programming is a mathematical technique that is used to achieve optimal results. To achieve optimal results, an objective function needs to satisfy the defined constraints. The feasible regions are computed for minimal energy dissipation and high network throughput.

### 5.1. Feasible Region Energy Minimization

For minimizing the energy consumption, we have defined the objective function in Equation (5).

(5) $$Minimum\Sigma_{r=1}^{r_{max}}E_{consumption}\left(r\right)\;\forall r\in r_{max}.$$

The linear constraints for energy minimization are given in Equations (5a)–(5c).

(5a) $$E_{trans},E_{rcv}\geq E_{re}$$

(5b) $$E_{trans},E_{rcv}\leq E_{init}$$

(5c) $$T_{rn}\leq T_{rmax}.$$

Equation (5a) shows restriction on transmission and receiving energy, which must not exceed the residual energy $E_{re}$ of the node; while transmission and receiving energy are restricted through Equation (5b) using initial energy $E_{init}$ of the node. Equation (5c) shows the restriction that for receiving a good quality signal, the sensed information should be transmitted within its given transmission range; where $T_{rn}$ is the transmission range of the node and $T_{rmax}$ is the maximum transmission range of the node; whereas, $E_{consumption}$ is the total energy consumed in data communication, i.e.,

(6) $$\Sigma_{r=1}^{r_{max}}E_{consumption}\left(r\right)=E_{trans}+E_{rcv}\;\forall r\in r_{max}.$$

where,

(7) $$E_{trans}=P_{trans}\left(\frac{Packet\_size}{Data\_rate}\right),$$

$E_{trans}$ is the transmission energy, and $P_{trans}$ is the transmission power.

(8) $$E_{rcv}=P_{rcv}\left(\frac{Packet\_size}{Data\_rate}\right).$$

$E_{rcv}$ is the receiving energy, and $P_{rcv}$ is the receiving power.

#### Graphical Analysis

For clear visualization of the objective function of energy minimization, graphical analysis is presented to compute all the values within the feasible region. Assuming $Packet_{size}=888$ bits, $Data_{rate}$ = 16,000 bps, $P_{trans}=\left\{12.5,25,\ldots ,50\right\}$ W and $P_{rcv}=\left\{0.0395,0.079,\ldots ,0.158\right\}$ W, the feasible solution for energy minimization is computed as:

(9) $$0.693\leq E_{tx}\leq 2.775$$

(10) $$0.002\leq E_{rx}\leq 0.0087$$

(11) $$0.695\leq E_{tx}+E_{rx}\leq 2.7837$$

**Figure 5:**
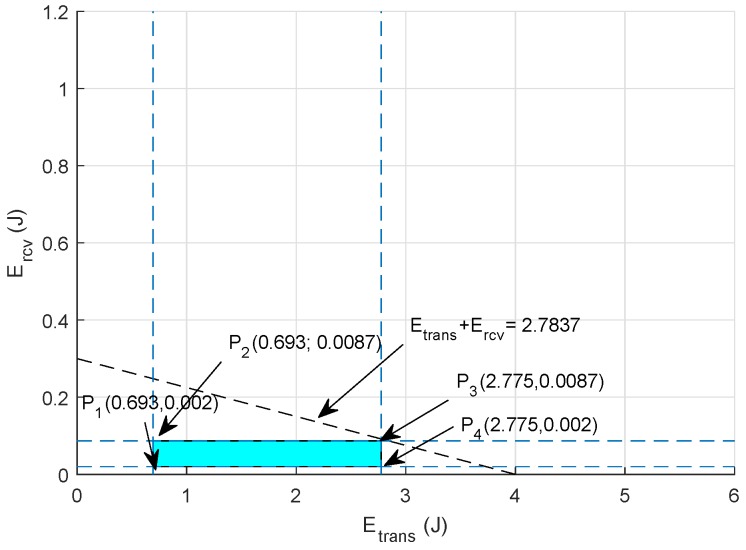
Feasible region: energy minimization.

The feasible region of energy minimization is shown in Figure 5 using points extracted from Equations (9)–(11), and the points on the boundary of this feasible region are:

$P_{1}\left(0.693,0.002\right)=0.695$ J
$P_{2}\left(0.693,0.0087\right)=0.7017$ J
$P_{3}\left(2.775,0.0087\right)=2.7837$ J
$P_{4}\left(2.775,0.002\right)=2.777$ J

Hence, selecting any value from these points results in minimum energy consumption in the network during communication.

### 5.2. Throughput Maximization

To maximize the throughput, we take the objective function along with its linear constraints to get the optimal results. The mathematical formulation is shown in Equation (12).

(12) $$Maximum\Sigma_{r=1}^{r_{max}}Thr\left(r\right)\;\forall r\in r_{max}.$$

Constraints of the objective function are given in (12a)–(13).

(12a) $$C_{1}:E_{tx},E_{rcv}\leq E_{i}$$

(12b) $$C_{2}:E_{tx}\leq E_{re}$$

(12c) $$C_{3}:TX_{n}\leq TX_{max}$$

(12d) $$C_{4}:D_{ij}\leq D_{ij}^{max}$$

(12e) $$C_{5}:Minimum\Sigma_{r=1}^{r_{max}}B_{Frw}^{r}.$$

Equation (12a) ensures that the energy required for transmission and reception should be less than the initial energy $E_{i}$ of the node. Equation (12b) shows the constraint that transmission energy $E_{tx}$ ought to be less than the residual energy $E_{re}$. Equation (12c) ensures that in order to receive a good quality signal, the data packet ought to be transmitted within its maximum transmission range $TX_{max}$; where $TX_{n}$ is the transmission range of the node and $TX_{max}$ is the maximum transmission range of the node. Equation (12d) maintains a threshold of distance between sender *i* and receiver *j* for successful communication. Equation (13) shows the restriction that the load on forwarder nodes and nodes that have less residual energy ought to be minimum; where $B_{Frw}$ is the bandwidth of forwarder nodes.

#### Graphical Analysis

Assuming a scenario where total bandwidth is between 2000 kHz and 4000 kHz, where $B_{Frw}$ shows the bandwidth allocated to the forwarding nodes with high residual energy and $B_{NFrw}$ is the bandwidth assigned to non-forwarding nodes, the bandwidth *B* allocated to $B_{Frw}$ and $B_{NFrw}$ is computed as follows using the aforementioned constraints in Equations (12a)–(12d):

(13) $$200\leq B_{Frw}\leq 1000$$

(14) $$2000\leq B_{NFrw}\leq 3000$$

(15) $$2200\leq B_{Frw}+E_{NFrw}\leq 4000$$

**Figure 6:**
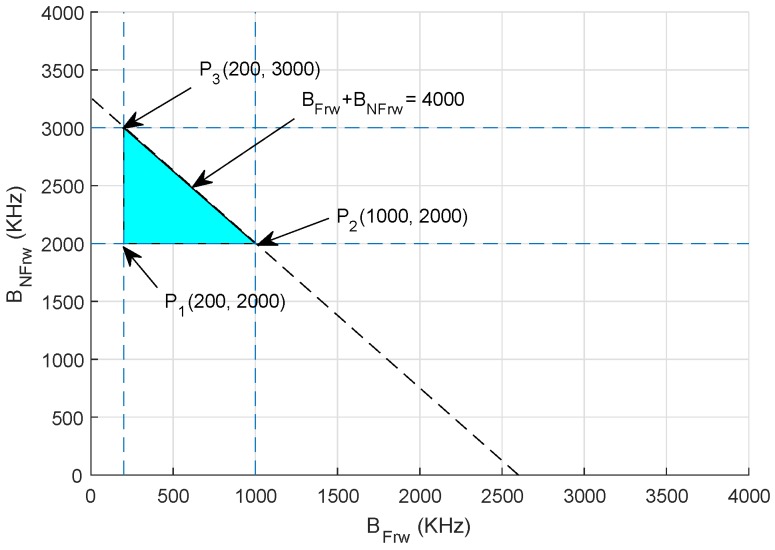
Feasible region: throughput maximization.

The feasible region is plotted in Figure 6, and points are extracted from Equations (13)–(15).

$P_{1}\left(200,2000\right)=2200$ kHz
$P_{2}\left(1000,2000\right)=3000$ kHz
$P_{3}\left(200,3000\right)=3200$ kHz

Thus, selecting any value from these points results in maximum network throughput.

## 6. Simulation Results

In this section, we evaluate the performance of the proposed schemes A-DBR, C-DBR, CA-DBR and B-DBR against the existing schemes: WDFAD-DBR [2] and Reliable and Energy-efficient Pressure-Based Routing (RE-PBR) [10].

### 6.1. Simulation Setup

In the simulations, we have used multi-sink architecture of dimensions 10 × 10 × 10 km${}^{3}$. The sensor nodes are randomly deployed in the given network field. The transmission range of a node is 2 km; the packet size is kept at 72 bytes; and the data rate is 16 kbps [4]. Each node starts with the initial energy of 100 J; where the consumption rate is 50 W during the transmission of data and 158 mW in receiving the packet. The node number varies from 100–500 during the execution of network operations. In order to handle the mobility of nodes, we have considered node speed in the horizontal direction 2 m/s. Additionally, the speed of the acoustic signal is 1500 m/s along with the bandwidth of 4 kHz. In a multi-sink architecture, we have deployed nine sinks at the surface of the water, which are housed with both acoustic and radio modems. The header size of the data packet is 11 bytes, and the ACKpacket is 50 bits. The simulations are conducted in Aqua-Sim (NS-2-based underwater sensor network simulator) to evaluate the performance of the proposed schemes against the baseline schemes. The aforementioned parameters are taken from [4] and are listed in Table 2.

### 6.2. Metrics

The objective of performing simulations is to evaluate the performance of our proposed schemes in terms of average Packet Delivery Ratio (PDR), energy tax, end-to-end delay and Accumulative Propagation Distance (APD). These metrics are defined as:

- Average PDR: It is defined as the total amount of data packets successfully received at the sink node to the total number of packets generated by the network nodes. It is calculated as: $PDR=\frac{Packets_{Received}}{Packets_{Transmitted}}$.
- Average energy tax: It is defined as the average energy consumption per node when a data packet is sent successfully to the sink. It is measured in joules (J). It is computed according to Equation (6).
- Average end-to-end delay: It is defined as the average time to transmit data from the source to the destination successfully. It is measured in seconds (s). The expression to find out complete path delay is: $\sum_{h=1}^{h_{max}}\frac{D(h_{i},h_{j})}{V}$. *h* is the hop count of nodes *i* and *j*. Vshows the speed of the acoustic signal, and $D(h_{i},h_{j})$ is the distance between node *i* and *j*.
- Average APD: It is the average accumulated propagation distance of all the data packets that are successfully sent to the sink nodes. It is measured in kilometers (km). The mathematical formulation is as follows: $\sum_{i}^{j}D\left(i,j\right)$.
- Network lifetime: It is the time period for which the network remained operational. It is measured in seconds (s). The mathematical expression is: $\sum_{t=1}^{t_{max}}NL\left(t\right)$, where NL shows the network lifetime in unit time (t).
- Packet drop ratio: It is defined as the ratio of the number of packets transmitted, however not delivered successfully at the destination node. It is formulated as 1−PDR.
- Alive nodes: It is the total number of nodes still alive after the termination of network operations. The mathematical expression is AN=N−DN, where AN is the number of alive nodes, DN denotes the quantity of dead nodes after complete battery depletion and *N* depicts the total number of nodes deployed in the network.

### 6.3. Performance Comparison

**Figure 7:**
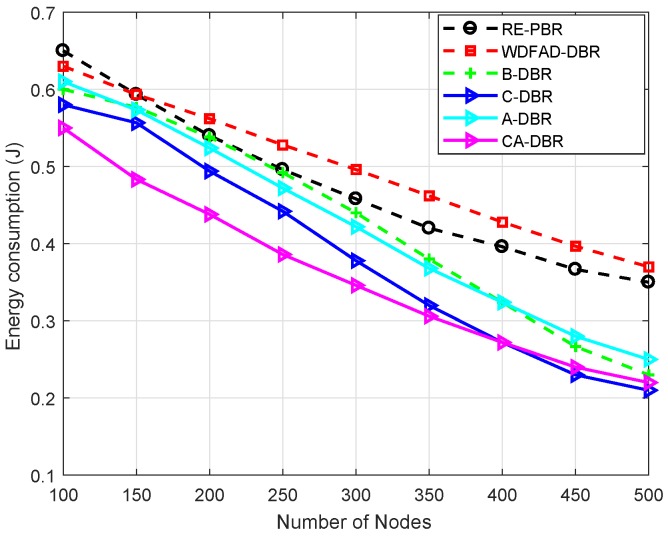
Comparison of energy tax. RE-PBR, Reliable and Energy-efficient Pressure-Based Routing; B-DBR, Backward transmission-based WDFAD-DBR.

**Figure 8:**
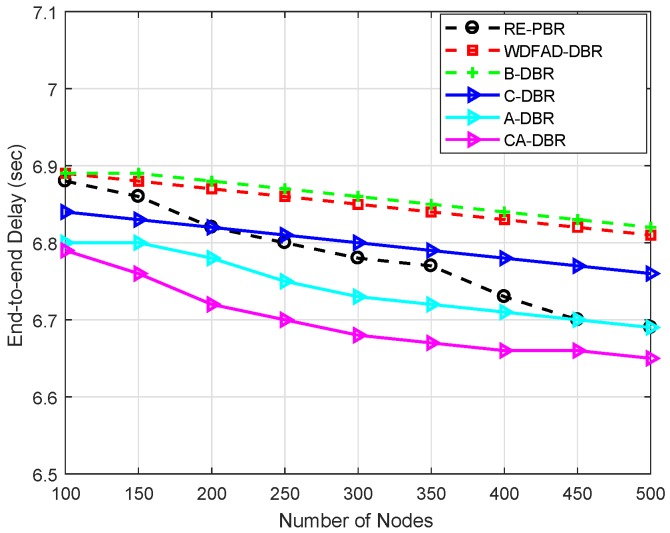
Comparison of end-to-end delay.

**Figure 9:**
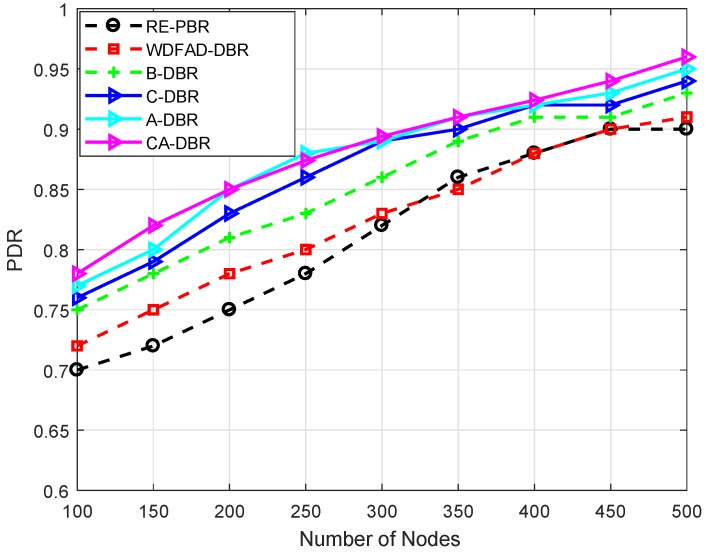
Comparison of Packet Delivery Ratio (PDR).

**Figure 10:**
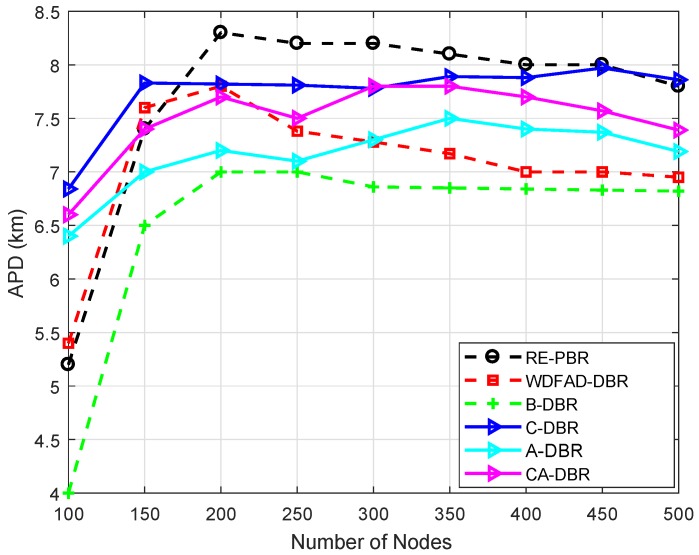
Comparison of Accumulative Propagation Distance (APD).

For performance evaluation, simulations are executed by comparing our proposed schemes with the WDFAD-DBR protocol. We evaluate our proposed schemes against WDFAD-DBR in terms of average PDR, energy tax, end-to-end delay and APD. The simulation results after comparison with WDFAD-DBR are shown in Figure 7, Figure 8, Figure 9 and Figure 10.

#### 6.3.1. Energy Tax

Figure 7 shows the energy tax of the baseline and proposed schemes. It clearly shows that as the density of nodes increases, it also increase the energy tax. Moreover, the high density of nodes also leads to more probability of data packet collision. This collision causes an increase in the packet drop ratio, which results in high energy consumption. The results in Figure 7 show that our schemes outperforms WDFAD-DBR in terms of energy tax. The proposed scheme A-DBR adjusts its transmission range adaptively when it finds no node in its range and continues to forward the data packet without any loss. In the proposed scheme C-DBR, the approach of clustering minimizes the transmission distance, directly affecting the energy consumption. In CA-DBR, fall back along with nomination of the forwarder node that has minimum number of neighbor nodes are selected. This also reduces the probability of packet loss and energy consumption.

The existing scheme WDFAD-DBR has high energy consumption due to high packet loss. Moreover, RE-PBR shows higher battery dissipation due to the longer routing path from the source to the destination in search of a high quality link. Initially, it has a greater dissipation rate compared to WDFAD-DBR when the node density is 100–150 because it is difficult to find a high link quality node. Furthermore, the battery utilization is almost the same at a 200-node density with B-DBR, but the more nodes get deployed in the network, the better the proposed scheme performs. This is only because when the node density increases, the number of backward transmissions becomes very less, thus the consumption rate decreases, as well.

The proposed scheme B-DBR uses one-hop backward transmission whenever it finds a void node. This scheme forwards the data packet towards the node that is located at a higher depth, and it has forwarders to forward the data packets towards the sink node. In a dense region, all protocols show almost similar behavior in terms of energy tax.

#### 6.3.2. End-to-End Delay

Figure 8 shows the end-to-end delay of the proposed schemes and the existing WDFAD-DBR scheme. A-DBR overcomes the void hole problem by adjusting its transmission range, which results in minimum packet drop and reduced end-to-end delay. In the proposed scheme C-DBR, the network is split into clusters to minimize the transmission distance, which helps with reducing the end-to-end delay. The CHs aggregate the sensed information and forward it towards the sink. The proposed scheme CA-DBR avoids collision, which reduces the end-to-end delay. The proposed scheme B-DBR uses backward transmission when it finds a void hole to continue data forwarding.

WDFAD-DBR has high end-to-end delay because it uses holding time, which increases its end-to-end delay. The proposed schemes outperform WDFAD-DBR, as void hole probability still exists in this protocol. The packet drop is due to void hole occurrence, which results in increased end-to-end delay in WDFAD-DBR.

End-to-end delay of of RE-PBR is moderate as compared to the proposed schemes B-DBR and C-DBR, whereas almost the same with A-DBR. This is because the aforesaid techniques use backtracking and cluster-based approaches, which take time to process the route from source to destination, while the later proposed scheme has the mechanism of transmission power adjustment instead of alternate route, which enables it to reduce delay.

#### 6.3.3. PDR

Figure 9 shows the PDR of the existing scheme WDFAD-DBR and our proposed schemes. In all schemes, PDR is increasing with the increase in node density. The reason for the increase in PDR is that void hole probability decreases with the increase in the density of nodes. The reason for less PDR of WDFAD-DBR is that this scheme only considers data transmission up to two hops, which does not eliminate the void hole occurrence. In WDFAD-DBR after two hops, a void hole may occur that will result in packet drop, which decreases the PDR. The PDR of WDFAD-DBR is slightly better than the RE-PBR because of better neighbor selection and holding time to avoid redundant transmissions, which lead to a better network lifetime and improved PDR than RE-PBR; whereas, RE-PBR is effective when the node density is 300–400; after that, its PDR again decreased because of high collision and higher packet drop rate.

In the proposed scheme A-DBR, if a void hole occurs after a two-hop transmission, then it adaptively adjusts its transmission range to find the forwarding neighbors and forward the packet towards the sink. In the second proposed scheme C-DBR, the network is divided into to clusters to further enhance the PDR and increase the network lifetime. Each cluster head is selected on the basis of residual energy, and cluster heads then communicate with one another to transmit the packets and adaptively adjust the transmission range, as well. In the proposed scheme CA-DBR, there are less packet drops, which results in increased PDR. In the proposed scheme B-DBR, instead of dropping the packet in the case of the void region, it uses backward transmission and forwards the data packet towards the base station. In WDFAD-DBR, it considers the current depth of the node and its expected next neighbor node depth; however, considering depth up to two hops is not the solution for void hole avoidance; thus, the PDR of WDFAD-DBR decreases.

#### 6.3.4. APD

Figure 10 shows that the APD of the existing scheme WDFAD-DBR is better than the proposed schemes, as it selects forwarders on the basis of depth of nodes. WDFAD-DBR has more APD, as it picks up the node with the minimum depth difference, and high priority is assigned through the calculated difference. If the forwarder node has neighbors, only then does it select it to relay the data. Therefore, a longer path is opted for, which is evident from Figure 10. On the other hand, the higher APD of RE-PBR is because of link quality, which leads to a longer data path, although the reliability of data delivery is high in RE-PBR as compared to the proposed and baseline schemes.

All the proposed schemes have increased APD as they avoid the shortest route to the sink to avoid collision and void holes. C-DBR performs better than A-DBR due to clustering in the network. Additionally, the transmission distance decreases, which ultimately increases the APD of the proposed scheme C-DBR. A-DBR adjusts the transmission range to reduce the void hole problem. Thus, the packet drop ratio decreases, which ultimately results in increased APD. The proposed schemes B-DBR and CA-DBR perform better than other algorithms as they are also selecting forwarders on the basis of depth along with backward transmission and collision avoidance.

#### 6.3.5. Alive Nodes

**Figure 11:**
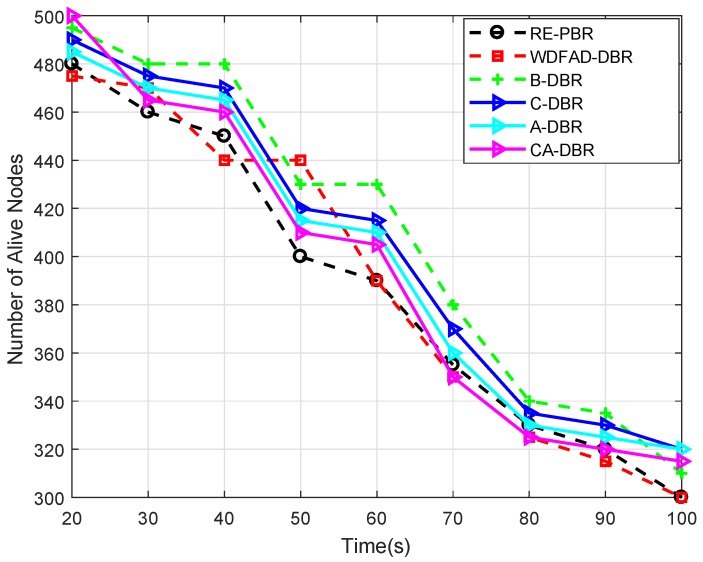
Comparison of alive nodes.

Figure 11 depicts the number of nodes alive in the network after a certain time period. At 20 s, the alive node number is different in each scheme, but remains almost within 470–500. However, the decrease is gradual in each scheme after a certain time period, which is obvious because of more and more data transmissions among the network nodes. WDFAD-DBR has a low number of alive nodes as it sends data to the neighbor node, which has further at least one more neighbor node. Thus, the immutable forwarder nomination leads to quick death of the node and creates a partition in the network; while RE-PBR has very strict criteria for neighbor selection based on the link quality, which also causes repeated selection and leads to void node creation. This void node creation is handled via A-DBR. CA-DBR, C-DBR and B-DBR all have almost the same alive node number.

#### 6.3.6. Network Lifetime

**Figure 12:**
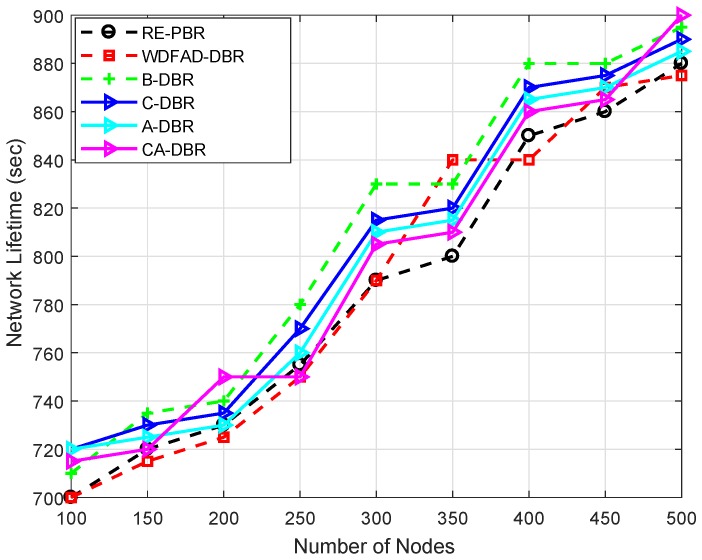
Comparison of network lifetime.

**Figure 13:**
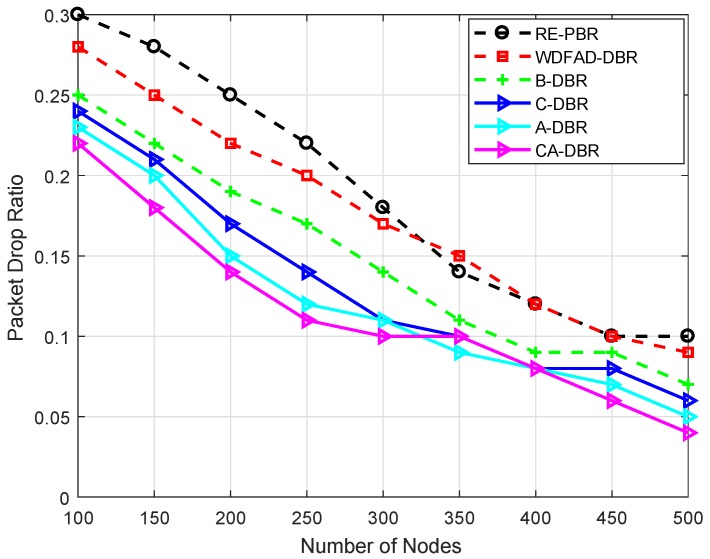
Comparison of the packet drop ratio.

Network lifetime is shown in Figure 12, which depends on the utilization of the node battery; if the dissipation of the node’s available power is efficient, then it will perform the network operation for a longer time. B-DBR leads in the lifetime plot compared to all the schemes. This scheme has a fall back recovery mechanism to ensure the data reach the destination. However, it does not find the data route all the time and drops the data packet; that is the reason it has a greater drop ratio than other proposed schemes, as given in Figure 13.

The WDFAD-DBR and RE-PBR have a greater drop ratio, but still, the lifetime is shorter, because, in-spite of dropping packets, the same node is elected again and again, until its death. The immutable destination node selection is due to the assignment of weight to forwarder nodes based on the link and depth difference in RE-PBR and WDFAD-DBR, respectively.

On the other hand, the other proposed schemes (A-DBR, CA-DBR and C-DBR) show almost the same behavior throughout the network lifespan. A-DBR has lower performance due to the adjustment of the transmission range, which consumes more battery and leads to a shorter network lifetime; while CA-DBR picks a collision-free path, which means a route with good quality gets selected time and again, leading to the sudden death of the intermediate nodes and creating a void hole. Thus, it reduces the network lifetime. On the other hand, C-DBR creates clusters to gather data packets locally and transmits a composite packet towards the destination. Thus, the repeated selection of a high energy node leads to the quick depletion of the node’s battery.

#### 6.3.7. Packet Drop Ratio

Figure 13 illustrates the number of packets dropped in the network. RE-PBR has a greater packet drop ratio when the node number is 100 because it explicitly chooses three metrics (energy, depth and link quality) to ensure a high quality signal reaches the destination. Although it ensures reliable data delivery, if the link quality is not good, it drops the data packet. This is the reason it has a high drop ratio.

WDFAD-DBR has a lower drop ratio as compared to RE-PBR because it looks up two hops and assigns priority to each neighbor node. Although it has a lower drop ratio, it is higher than all proposed schemes.

The proposed schemes show variation in the results with respect to each other and have better performance than RE-PBR and WDFAD-DBR. The CA-DBR has the minimum drop rate compared to the baseline and other proposed schemes. The reason is the consideration of collision on the wireless channel. It chooses the link that has the minimum number of nodes surrounding the source node. This parameter helps with delivering the data successfully at the destination. Whereas C-DBR and A-DBR have a similar pattern of drop ratio, A-DBR adjusts the transmission range to bypass the hot spot problem; although, sometimes, it depletes energy suddenly in transmission adjustment; while C-DBR is a cluster based scheme and transmits the composite data packet, which means, if a composite packet of 10 packets is dropped, it would be considered as one. The B-DBR uses a backtrack process to find an alternate path instead of dropping the data packet; therefore, it has a lower drop ratio than RE-PBR and WDFAD-DBR.

## 7. Performance Trade-Offs

In this section, we review the performance of our proposed schemes A-DBR, C-DBR, CA-DBR and B-DBR with the existing base scheme WDFAD-DBR. A-DBR reduces void nodes by adaptively adjusting the transmission range and achieves reduced energy consumption in dense regions; while WDFAD-DBR makes a routing decision based on the weighting sum of depth difference up to two nodes. Therefore, the possibility of confronting a void hole still exists, which results in packet loss and high end-to-end delay. C-DBR has reduced end-to-end delay at the cost of high energy consumption as compared to A-DBR due to clustering. CA-DBR experienced less consumption of energy and low end-to-end delay at the cost of high APD. B-DBR is able to minimize the void node probability, which results in high PDR and reduced end-to-end delay at the cost of high APD. Other proposed schemes have increased APD as they avoid the shortest route to the sink in order to avoid collision and void holes. The summary of simulation results is presented in Table 3 and the trade-offs are shown in Table 4.

## 8. Conclusions

In this paper, we proposed routing protocols that are energy efficient, reliable and show improved network lifetime. The first scheme A-DBR reduced the void nodes’ occurrence by adaptively adjusting the transmission range. Additionally, it achieved minimum energy consumption by avoiding data loss in the network. In the second scheme C-DBR, end-to-end delay is reduced at the cost of high energy consumption. The third scheme B-DBR minimized the void node occurrence probability along with a high packet delivery ratio. However, the cost of high accumulative propagation distance is paid. The fourth scheme CA-DBR experienced less energy consumption along with low end-to-end delay. Simulation results show the effectiveness of the proposed schemes in terms of average packet delivery ratio, average energy tax and average end-to-end delay.

## Figures and Tables

**Table 1:** Summary of UWSN routing schemes discussed in related work. AHH-VBF, Adaptive Hop-by-Hop Vector-Based Forwarding; HydroCast, Hydraulic-pressure-based anyCast; DBR, Depth-Based Routing; iAMCTD, improved Adaptive Mobility of Courier nodes in Threshold-optimized DBR; E-CARP, Energy-efficient Channel-Aware Routing Protocol; ARCR, Adaptive Relay Chain Routing.

Technique	Features	Achievements	Limitations
AHH-VBF [1]	Location-aware routing protocol, concept of adaptive virtual pipeline	Reduced duplicate packets and unnecessary energy consumption is avoided	Void hole problem exists
GEDAR [15]	GEographic and opportunistic routing with Depth Adjustment-based topology control for communication	Void hole avoidance results in increased performance of the network	High energy consumption and high end-to-end delay
HydroCast [16]	Pressure-based routing protocol and efficient anycast routing algorithm	Improved packet delivery ratio	Low performance and increased energy consumption
H2-DARP-PM [17]	Hop-by-Hop Dynamic Addressing-based routing protocol for Pipeline Monitoring	Improved packet delivery ratio	High energy consumption
Delay-sensitive schemes [18]	Improved delay-sensitive versions, adaptable to time-critical applications	Minimize end-to-end delay and improve performance and network lifetime	Duplication of packets occurs, high energy consumption and void hole problem exists
ACH${}^{2}$ [28]	Free association mechanism where nodes associate with CHs	Minimizing energy consumption and enhances network lifetime	Transmission delay
FSO and EM wave-based communication schemes [19]	Free Space Optical and electromagnetic wave-based communication schemes	Reduced energy consumption	High end-to-end delay
CBSST [20]	Cluster-Based Sleep/wakeup Scheduling Technique for WSN	Reduced energy consumption, enhanced network lifetime and packet delivery ratio	Keeping the same CH throughout the network lifetime causes problems for network lifetime
UCBNL [21]	A high efficiency Uneven Cluster deployment algorithm Based on Network Layered for event coverage in UWSNs	Enhanced packet delivery ratio, less energy consumption and improved network lifetime	Irregular clustering causes alteration in the network
PSO-ECHS [22]	Energy-efficient CH Selection that is based on particle swarm optimization	Energy efficiency achieved	Only for homogeneous networks
EDDEEC [29]	Enhanced Developed Distributed Energy-Efficient Clustering	Shows improved performance in terms of stability period, network lifetime and packet delivery ratio.	Imbalanced clustering and reelection increases overhead
Energy-efficient routing protocol [23]	SEEC, CSEEC and CDSEEC for UWSNs	Reduced energy consumption	Low packet delivery ratio
DBR [24]	Handles dynamic networks efficiently, requires only local depth information and greedy forwarding	Improved network lifetime and packet delivery ratio	Void holes, increased energy consumption and high end-to-end delay
iAMCTD [25]	Location-free routing protocol specially designed for time-critical applications	Improved network lifetime, minimized end-to-end delay	Void holes still exist & overhead due to control packets’ exchange
E-CARP [26]	Distributed cross-layer reactive protocol, important for sensory data collection and transmission	Improved network lifetime and reduced energy consumption	Reduced throughput and high path loss due to mobility
ARCR [27]	Network is divided into clusters and mobile nodes used to collect data from other sensor nodes and forward them to the sink	Achieves energy efficiency, maximum network lifetime and load balancing	Network disconnects when the relay nodes are disorganized

**Table 2:** Simulation parameters.

Parameter	Value
Nodes	100–500
Sinks	9
Network Dimensions (km${}^{3}$)	10 × 10 × 10
Movement Speed of Nodes (m/s)	2
Acoustic Propagation Speed (m/s)	1500
Initial Energy (J)	100
Transmission Range (km)	2
Transmission Power (dB reμPa)	90
Total Bandwidth (kHz)	4
Sending Energy (W)	50
Receiving or Idle Energy (mW)	158
Header Size (bytes)	11
Payload (bytes)	72
Data Rate (kbps)	16
Size of ACK (bits)	50

**Table 3:** Summarized simulation results.

Parameters	RE-PBR	WDFAD-DBR	B-DBR	A-DBR	C-DBR	CA-DBR
Network lifetime	88%	87%	89%	85%	88.4%	90%
End-to-end delay	15%	09%	10%	13.6%	14.6%	14.8%
APD	22%	30%	35.8%	21%	27%	26%
PDR	90%	91.5%	93%	94.2%	95%	96%
Alive nodes	60%	60%	62%	64%	63.3%	64%
Packet drop ratio	10%	8.5%	7%	5.8%	5%	4%
Energy consumption	38%	39%	24%	21%	26%	22%

**Table 4:** Performance trade-offs.

Schemes	Features	Achieved Parameters	Trade-Offs
WDFAD-DBR [4]	Routing based on depth and energy	Less packet drops and improved network lifetime, decreased APD	High energy consumption and high end-to-end delay
RE-PBR [10]	Routing based on link quality, depth and energy	Improved network lifetime and low delay	High packet drop ratio and more APD
A-DBR	Routing based on depth and energy along with transmission range adjustment	Void hole avoidance results in increased performance of the network and reduced energy consumption	High energy consumption in sparse regions and increased APD
C-DBR	Routing based on depth and energy along with clustering	Improved PDR, low end-to-end delay and APD	Increased energy consumption due to clustering compared to A-DBR
CA-DBR	Routing based on depth and energy with collision avoidance	Reduced energy consumption and low delay	Increased APD
B-DBR	Routing based on depth and energy with tracking features	High PDR and reduced delay	Increased APD

## References

[1] Yu H., Yao N., Liu J. (2015). An adaptive routing protocol in underwater sparse acoustic sensor networks. Ad Hoc Netw..

[2] Coutinho R.W., Boukerche A., Vieira L.F., Loureiro A.A. (2018). Underwater Wireless Sensor Networks: A New Challenge for Topology Control-Based Systems. ACM Comput. Surv..

[3] Coutinho R.W.L., Boukerche A., Vieira L.F.M., Loureiro A.A.F. (2015). A novel void node recovery paradigm for longterm underwater sensor networks. Ad Hoc Netw..

[4] Yu H., Yao N., Wang T., Li G., Gao Z., Tan G. (2016). WDFAD-DBR: Weighting depth and forwarding area division DBR routing protocol for UASNs. Ad Hoc Netw..

[5] Jiang S. (2018). On reliable data transfer in underwater acoustic networks: A survey from networking perspective. IEEE Commun. Surv. Tutor..

[6] Kheirabadi M.T., Mohamad M.M. (2013). Greedy routing in underwater acoustic sensor networks: A survey. Int. J. Distrib. Sens. Netw..

[7] Li N., Martínez J.F., Chaus J.M.M., Eckert M. (2016). A survey on underwater acoustic sensor network routing protocols. Sensors.

[8] Han G., Jiang J., Bao N., Wan L., Guizani M. (2015). Routing protocols for underwater wireless sensor networks. IEEE Commun. Mag..

[9] Mitra S., Roy A. (2015). Communication void free routing protocol in wireless sensor network. Wirel. Pers. Commun..

[10] Khasawneh A., Latiff M.S.B.A., Kaiwartya O., Chizari H. (2018). A reliable energy-efficient pressure-based routing protocol for underwater wireless sensor network. Wirel. Netw..

[11] Farhan L., Kharel R., Kaiwartya O., Hammoudeh M., Adebisi B. (2018). Towards green computing for Internet of things: Energy oriented path and message scheduling approach. Sustain. Cities Soc..

[12] Kaiwartya O., Abdullah A.H., Cao Y., Lloret J., Kumar S., Shah R.R., Prasad M., Prakash S. (2018). Virtualization in wireless sensor networks: Fault tolerant embedding for internet of things. IEEE Internet Things J..

[13] Aliyu A., Abdullah A.H., Kaiwartya O., Cao Y., Lloret J., Aslam N., Joda U.M. (2018). Towards video streaming in IoT Environments: Vehicular communication perspective. Comput. Commun..

[14] Faheem M., Tuna G., Gungor V.C. (2017). LRP: Link quality aware queue based spectral clustering routing protocol for underwater acoustic sensor networks. Int. J. Commun. Syst..

[15] Coutinho R.W., Boukerche A., Vieira L.F., Loureiro A.A. (2016). Geographic and opportunistic routing for underwater sensor networks. IEEE Trans. Comput..

[16] Noh Y., Lee U., Lee S., Wang P., Vieira L.F., Cui J.H., Gerla M., Kim K. (2016). Hydrocast: Pressure routing for underwater sensor networks. IEEE Trans. Veh. Technol..

[17] Abbas M.Z., Bakar K.A., Ayaz M., Mohamed M.H., Tariq M. (2017). Hop-by-Hop Dynamic Addressing Based Routing Protocol for Monitoring of long range Underwater Pipeline. KSII Trans. Internet Inf. Syst..

[18] Javaid N., Jafri M.R., Ahmed S., Jamil M., Khan Z.A., Qasim U., Al-Saleh S.S. (2015). Delay-sensitive routing schemes for underwater acoustic sensor networks. Int. J. Distrib. Sens. Netw..

[19] Yadav S., Kumar V. (2017). Optimal Clustering in Underwater Wireless Sensor Networks: Acoustic, EM and FSO Communication Compliant Technique. IEEE Access.

[20] Sasikala V., Chandrasekar C. (2013). Cluster based Sleep/Wakeup Scheduling Technique for WSN. Int. J. Comput. Appl..

[21] Yu S., Liu S., Jiang P. (2016). A High-Efficiency Uneven Cluster Deployment Algorithm Based on Network Layered for Event Coverage in UWSNs. Sensors.

[22] Rao P.S., Jana P.K., Banka H. (2017). A particle swarm optimization based energy efficient cluster head selection algorithm for wireless sensor networks. Wirel. Netw..

[23] Sher A., Javaid N., Azam I., Ahmad H., Abdul W., Ghouzali S., Niaz I.A., Khan F.A. (2017). Monitoring square and circular fields with sensors using energy-efficient cluster-based routing for underwater wireless sensor networks. Int. J. Distrib. Sensor Netw..

[24] Yan H., Shi Z.J., Cui J.H. (2008). DBR: Depth-based routing for underwater sensor networks. Networking 2008 Ad Hoc and Sensor Networks, Wireless Networks, Next Generation Internet.

[25] Javaid N., Jafri M.R., Khan Z.A., Qasim U., Alghamdi T.A., Ali M. (2014). Iamctd: Improved adaptive mobility of courier nodes in threshold-optimized dbr protocol for underwater wireless sensor networks. Int. J. Distrib. Sens. Netw..

[26] Zhou Z., Yao B., Xing R., Shu L., Bu S. (2015). E-CARP: An energy efficient routing protocol for UWSNs in the internet of underwater things. IEEE Sens. J..

[27] Kong L., Ma K., Qiao B., Guo X. (2016). Adaptive relay chain routing with load balancing and high energy efficiency. IEEE Sens. J..

[28] Ahmad A., Javaid N., Khan Z.A., Qasim U., Alghamdi T.A. (2014). (*ACH*)^2^: Routing Scheme to Maximize Lifetime and Throughput of Wireless Sensor Networks. IEEE Sens. J..

[29] Yildiz H.U., Gungor V.C., Tavli B. (2018). Packet Size Optimization for Lifetime Maximization in Underwater Acoustic Sensor Networks. IEEE Trans. Ind. Inform..

[30] Bu R., Wang S., Wang H. (2018). Fuzzy logic vector-based forwarding routing protocol for underwater acoustic sensor networks. Trans. Emerg. Telecommun. Technol..

[31] Khalid M., Cao Y., Ahmad N., Khalid W., Dhawankar P. (2018). Radius-based multipath courier node routing protocol for acoustic communications. IET Wirel. Sens. Syst..

